# Extracellular matrix proteins (fibronectin, collagen III, and collagen I) immunoexpression in goat tuberculous granulomas (*Mycobacterium caprae*)

**DOI:** 10.1007/s11259-022-09996-3

**Published:** 2022-09-22

**Authors:** Carlos Neila, Agustín Rebollada-Merino, Javier Bezos, Lucía de Juan, Lucas Domínguez, Antonio Rodríguez-Bertos

**Affiliations:** 1 VISAVET Health Surveillance Centre, Complutense University of Madrid, 28040 Madrid, Spain; 2 Department of Internal Medicine and Animal Surgery, Faculty of Veterinary Medicine, Complutense University of Madrid, 28040 Madrid, Spain; 3 Department of Animal Health, Faculty of Veterinary Medicine, Complutense University of Madrid, 28040 Madrid, Spain

**Keywords:** Collagen, Fibronectin, Fibrosis, *Mycobacterium caprae*, Tuberculous granuloma

## Abstract

The lesion resulting from the interaction between *Mycobacterium* and the host immune response is the tuberculous granuloma. Tuberculous granulomas, except in incipient stages, are partially or totally encapsulated by connective tissue. The aim of this study was to assess the immunoexpression of the extracellular matrix proteins fibronectin, collagen III, and collagen I in granulomas caused by *Mycobacterium caprae* in goats (*Capra aegagrus hircus*) to understand capsule development at different granuloma stages. For this purpose, a retrospective study of 56 samples of tuberculous granulomas in lung (*n* = 30) and mediastinal lymph node (*n* = 26) from 17 goats naturally infected with *M. caprae* in stages I (*n* = 15), II (*n* = 14) and III (*n* = 27) was carried out. Fibronectin immunoreaction was extracellular, fibrillar-reticular in the center of stage I, II and III granulomas and peripheral in stages II and III granulomas. Collagen III immunoexpression was extracellular and fibrillar in the center of stages I, II and III tuberculous granulomas in lung and mediastinal lymph node, and progressive expression was observed in the periphery of stages II and III granulomas. Finally, collagen I immunoexpression was extracellular and fibrillar, showing a progressive loss of central expression and an increase in peripheral expression in stage III granulomas compared to stage I granulomas. Immunoexpression of these extracellular matrix proteins could help understand fibrogenesis and dating in tuberculous granuloma in both animal models and humans.

**Supplementary Information:**

The online version contains supplementary material available at 10.1007/s11259-022-09996-3.

## Introduction

Tuberculosis (TB) is a zoonosis caused by species belonging to the *Mycobacterium tuberculosis* complex (MTBC), particularly *M. tuberculosis*, *M. bovis*, and *M. caprae* (Malone and Gordon 2017). The worldwide distribution and wide host range makes TB a persistent threat to both human and animal health. In 2019, it was estimated that 10 million people contracted TB and 1.4 million died as a result (World Health Organization 2020). TB in livestock has important public health implications and leads to considerable economic losses and social impact.

*M. caprae* was given species status in 2003 because of its biochemical and epidemiological characteristics (Aranaz et al. 2003). It has been isolated from different species: goat (*Capra aegagrus hircus*), domestic cattle (*Bos primigenius taurus*), domestic pig (*Sus scrofa domesticus*) and wild species. In animals, MTBC infection is mainly aerogenous, although digestive, genital, transplacental or intramammary routes have also been described (Domingo et al. 2014). The primary respiratory complex is established in aerogenous primary infections, and can be complete if lung and draining lymph node are affected, or incomplete if lesions are present only in the lymph node (Domingo et al. 2014). The goat has been suggested as an animal model of human TB (Pérez de Val et al. 2011).

The most characteristic lesion resulting from the interaction between MTBC and the host immune response is the tuberculous granuloma, a highly complex and dynamic lesion (Warsinske et al. 2017). The ultimate function of these compact and hypoxic lesions is to contain and neutralize mycobacterial infection. The structure of granulomas varies depending on the MTBC species involved, the course of infection, and the host and its individual immune response. The specific histopathological features of MTBC lesions plus ancillary acid-fast stains and MTC immunohistochemistry, contribute to increase speed in the postmortem diagnosis of TB in animals and humans (Orme and Basaraba 2014).

Tuberculous granuloma capsule formation is an emerging field of study due to its potential application in the investigation of anti-fibrotic therapies in human TB (Evans et al. 2020). Tuberculous granulomas in cattle can be histopathologically classified into different stages: I, II, III and IV (Wangoo et al. 2005). Granulomas are totally or partially encapsulated by connective tissue during fibrosis, except in the incipient stages. Fibrosis is a pathological process that modifies tissue architecture and is characterized by the deposition of extracellular matrix proteins mainly produced by fibroblasts and myofibroblasts (Hortle and Oehlers 2020). Fibrosis occurs when the physiological processes of injury and repair are dysregulated by intense, repetitive, or chronic tissue damage. Granuloma encapsulation has several functions, such as containing the infection, isolating and preventing mycobacteria dissemination, and delimiting the lesion from healthy tissue (Shkurupiy et al. 2014; Warsinske et al. 2017). It also contributes to nutrient deprivation, thereby limiting mycobacterial proliferation. However, the capsule also prevents the diffusion of antimicrobial agents inside the lesion, thus favoring the survival of bacilli inside the granuloma and limiting therapeutic success (Tsenova and Singhal 2020). This may facilitate dissemination and progression of the disease (Tsenova and Singhal 2020).

The main extracellular matrix proteins that contribute to fibrosis are fibronectin, collagen III, and collagen I. Fibronectin is a glycoprotein with functions of cell adhesion, migration, proliferation, and apoptosis that mediates fibrosis (Patten and Wang 2020). Fibronectin expression has been described in the periphery of granulomas and its synthesis is related to macrophages and fibroblasts (Marshall et al. 1996). Fibronectin-specific binding sites for collagen facilitate the deposition of the definitive extracellular matrix.

Collagen III is abundant in granulation tissue and is subsequently replaced by collagen I (Patten and Wang 2020). Fibroblast procollagen synthesis is mediated by the macrophage-mediated production of transforming growth factor β (TGF-β) in bovine TB (Canal et al. 2017). Immunohistochemical studies have described increased expression of TGF-β1 in stages III and IV bovine granulomas (Canal et al. 2017). Procollagen III expression has been observed in the periphery of human lung granulomas (Kaarteenaho-Wiik et al. 2007).

The most common extracellular matrix component is collagen I (DiFazio et al. 2016). Procollagen I has been described in stage III and IV bovine tuberculous granulomas (Wangoo et al. 2005). The expression of procollagen I inside and at the periphery of granulomas has been previously observed in pulmonary (Kaarteenaho-Wiik et al. 2007) and cutaneous (Marshall et al. 1996) TB in humans. In tuberculous granulomas, the renewal of collagen is regulated by metalloproteinases and metalloproteinase inhibitors, which are synthesized mainly by macrophages or fibroblasts.

The purpose of this study was to assess the immunoexpression of extracellular matrix proteins (fibronectin, collagen III, and collagen I) with a view to understand the fibrosis dynamics in in caprine tuberculous granulomas at different stages.

## Materials and methods

### Samples

Goat tuberculous granulomas from lung and mediastinal lymph node samples submitted for diagnosis TO the Pathology and Veterinary Forensic Medicine Unit of the VISAVET Health Surveillance Centre (Complutense University of Madrid) during 2017 were retrospectively screened and included in this study. All goats were older than 8 weeks of age. *M. caprae* infection was confirmed by liquid culture (Lowenstein-Jensen) and PCR, as described elsewhere (Roy et al. 2019).

### Histological processing

Tissue samples collected during necropsy were fixed 24 hours, trimmed, dehydrated (Citadel 2000 Tissue Processor, Thermo Fisher Scientific, Waltham, MA), embedded in paraffin (Histo Star Embedding Workstation, Thermo Fisher Scientific), sectioned (Finesse ME + Microtome, Thermo Fisher Scientific) and stained with hematoxylin-eosin (HE) (Gemini AS Automated Slide Stainer, Thermo Fisher Scientific). Finally, samples were mounted in glass slides (CTM6 Coverslipper, Thermo Fisher Scientific). HE-stained sections were evaluated and classified according to a published staging of granulomas in *M. bovis*-infected cattle (Wangoo et al. 2005): stage I (initial), II (solid), and III (minimal necrosis). Stage IV granulomas were not included due to their structural similarity to stage III granulomas, despite a larger size and multicentric nature.

### Immunohistochemistry

The immunohistochemical study was performed using a commercial kit (ImmPRESS®-VR Horse Anti-Rabbit IgG Polymer Kit, Vector Laboratories LTD, Peterborough, UK). The slides were de-paraffinized in xylene (Casa Álvarez Material Científico SA, Madrid, Spain) and rehydrated in ethanol series. After thermic antigen retrieval using a pressure cooker with citrate buffer pH 6 (Panreac Química SLU), the samples were incubated in a hydrogen peroxide solution in methanol (Panreac Química SLU) to inactivate the endogenous peroxidase. The samples were then incubated in horse serum (Vector Laboratories), the excess serum was removed, and the diluted primary antibody was added (Table 1). For negative controls, the primary antibody was replaced by a commercially-available universal negative control reagent (Enzo Life Sciences, Farmingdale, NY) (Ramos-Vara et al. 2008) (Supplementary Fig. 1). Goat-human collagens I and III, and fibronectin homology was performed using pBLAST tool (Bethesda, MD) showing over 92% of protein identity. A commercial kit was used to reveal the samples (ImmPACT® NovaRED™ Peroxidase Substrate, Vector Laboratories LTD). Finally, samples were counterstained with hematoxylin (Gemini AS Automated Slide Stainer, Thermo Fisher Scientific) and mounted (CTM6 Coverslipper, Thermo Fisher Scientific).

**Table 1:** Antibodies employed, species reactivity, dilution, incubation conditions, and source

Antibody	Species reactivity	Dilution	Incubation	Source
Rabbit anti-fibronectin polyclonal (IgG)	Mouse, rat, hamster, cow, dog, human, African green monkey, Chinese hamster, Syrian hamster	1:200	2 hours, 20 °C	Thermo Fisher Scientific
Rabbit anti-collagen III polyclonal (IgG)	Human, mouse, rat	1:200	1 hour, 20 °C	Wuhan Fine Biotech
Rabbit anti-collagen I polyclonal (IgG)	Mouse, rat, goat, horse, cow, human, pig, common marmoset	1:100	24 hours, 4 °C	Abcam

### Evaluation

Glass slides obtained were evaluated under light microscopy by a senior veterinary pathologist (A.R.-B.) and two veterinary pathologists in training (C.N.-M. and A.R.-M.). The detection of extracellular matrix proteins in internal structures was used as a positive control. Fibronectin physiological expression in the lung is in peribronchial connective tissue and interstitium of alveolar walls (Maxie 2015). Collagen I and III physiological expression in the lung is in peribronchial connective tissue, interstitium of alveolar walls, adventitia, and visceral pleura (Konomi et al. 1981). The images were obtained using a digital camera (MC170 HD, Leica, Wetzlar, Germany) connected to an optical microscope (DM2000, Leica) using a commercial software (Leica Application Suite, version 4.6.0, Leica).

## Results

Fifty-six tuberculous granulomas from lung (30/56; 53.6%) and mediastinal lymph node (26/56; 46.4%) samples from 17 goats were individually evaluated. Stages I (15/56; 26.8%), II (14/56; 25.0%) and III (27/56; 48.2%) granulomas in lung and mediastinal lymph nodes were included in the study. Several granulomas with the same or different stages of evolution could be observed in the same histological section in some cases.

### Stage I granulomas

**Fig. 1:**
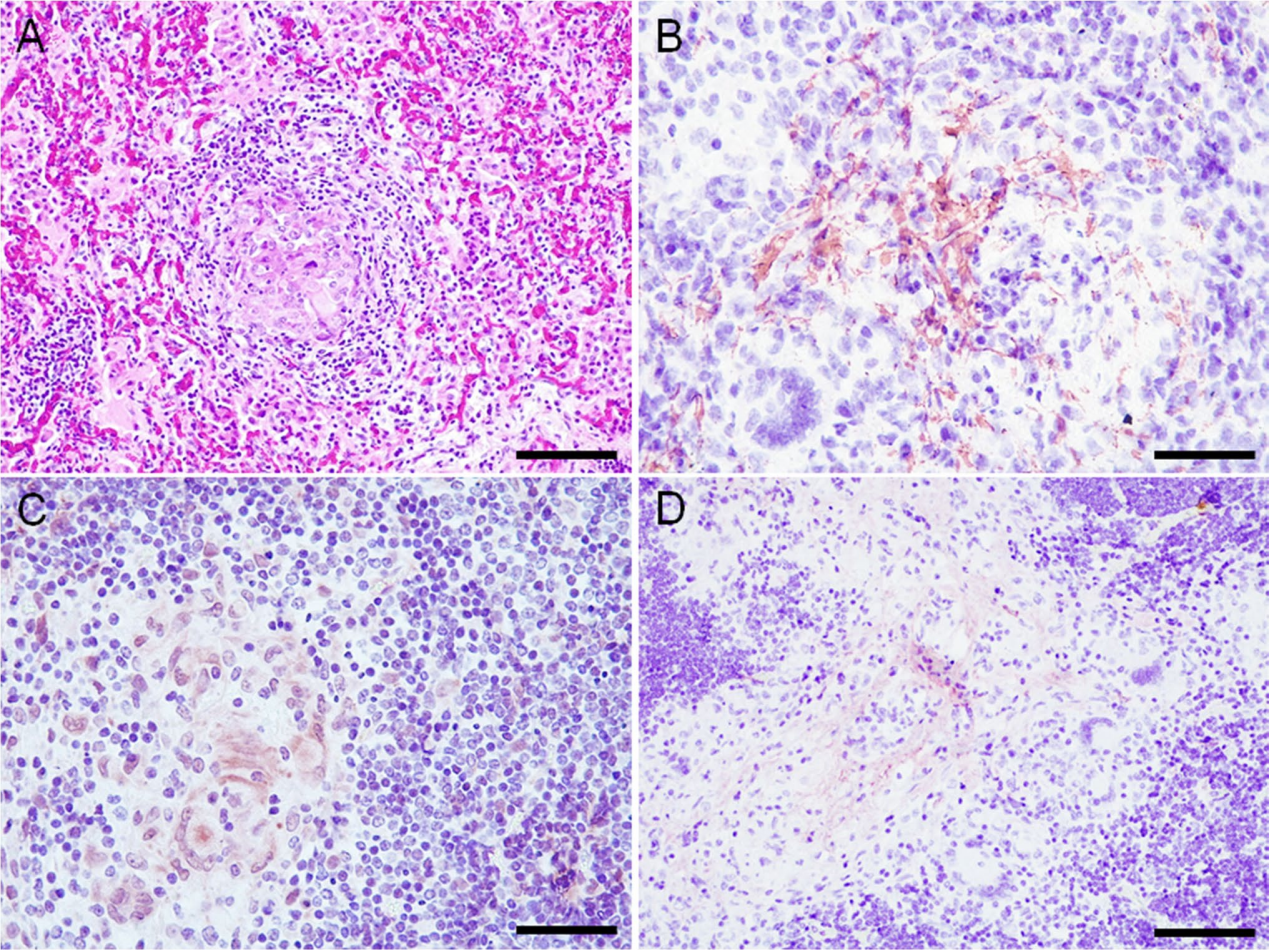
**A**-**D** Stage I granulomas (Wangoo et al. 2005) in goat (*Capra aegagrus hircus*) naturally infected with *Mycobacterium caprae*. **A**) Lung: chronic multifocal granulomatous pneumonia. HE, scale bar: 200 μm; **B**) Lung: extracellular, fibrillar-reticular, intense fibronectin immunoexpression in the centre of granulomas. Rabbit polyclonal anti-fibronectin antibody, scale bar: 100 μm; **C**) Lymph node: extracellular and cytoplasmic (epithelioid cells and Langhans-type multinucleated giant cells), moderate type III collagen immunoexpression in the centre of granulomas. Rabbit polyclonal anti-type III collagen antibody, scale bar: 100 μm; **D**) Lymph node: extracellular, fibrillar, mild type I collagen immunoexpression in the centre of granulomas. Rabbit polyclonal anti-type I collagen antibody, scale bar: 200 μm

Stage I granulomas studied (15/56; 26.8%) were located in the lung (8/15; 53.3%) and mediastinal lymph node (7/15; 46.7%). Regardless of the location, they were characterized as small, round, irregular, compact, well demarcated, and non-encapsulated lesions (Fig. 1A). They were composed of numerous epithelioid cells: medium-sized, round cells with a pale eosinophilic cytoplasm and a central, oval, basophilic nucleus with dispersed chromatin. Lymphocytes, and rarely neutrophils and Langhans-type multinucleated giant cells, were occasionally observed.

Fibronectin immunoexpression was extracellular and fibrillar-reticular in the center of the lesion in all stage I granulomas in lung (8/8; 100%) and in most mediastinal lymph nodes (6/7; 85.7%) (Fig. 1B). Fibronectin immunoexpression was observed in the periphery of the lesion in only 1 stage I granuloma in lung (1/8; 12.5%) and was not observed in the periphery of mediastinal lymph node granulomas (0/7; 0.0%) (Table 2). Diffuse, cytoplasmic expression was occasionally observed in some epithelioid cells in stage I granulomas in lung (3/8; 37.5%) and mediastinal lymph node (4/7; 57.1%) (Suppl. Table 1).

**Table 2:** Immunohistochemical results of fibronectin, type III collagen and type I collagen in stage I, II and III granulomas (Wangoo et al. 2005) in goat (*Capra aegagrus hircus*) naturally infected with *Mycobacterium caprae*

Granuloma stage	Immunoexpression	Fibronectin		Type III collagen		Type I collagen	
		Lung	Lymph node	Lung	Lymph node	Lung	Lymph node
Stage I	Central	8/8 (100%)	6/7 (85.7%)	8/8 (100%)	3/3 (100%)	7/8 (85.7%)	3/3 (100%)
	Peripheral	1/8 (12.5%)	0/7 (0.0%)	0/8 (0.0%)	2/3 (66.7%)	0/8 (0.0%)	0/3 (0.0%)
Stage II	Central	8/8 (100%)	5/6 (83.3%)	7/7 (100%)	5/5 (100%)	6/7 (85.7%)	5/6 (83.3%)
	Peripheral	3/8 (37.5%)	4/6 (66.7%)	5/7 (71.4%)	5/5 (100%)	1/7 (14.3%)	4/6 (66.7%)
Stage III	Central	13/14 (92.9%)	13/13 (100%)	6/9 (66.7%)	11/11 (100%)	2/9 (22.2%)	2/12 (16.7%)
	Peripheral	14/14 (100%)	13/13 (100%)	9/9 (100%)	11/11 (100%)	9/9 (100%)	11/12 (91.7%)

Immunoexpression of collagen III in stage I granulomas was extracellular and fibrillar in the center of the lesion in lung (8/8; 100%) and mediastinal lymph nodes (3/3; 100%) (Fig. 1C). Collagen III immunoexpression was not observed in the periphery of the lesion in any stage I granuloma in lung (0/8; 0.0%) but was positive in some mediastinal lymph nodes (2/3; 66.7%) (Table 2). Procollagen III expression in the cytoplasm of epithelioid cells and Langhans-type multinucleated giant cells was observed diffusely in the granulomas in lung (7/8; 87.5%) and mediastinal lymph nodes (3/3; 100%) (Suppl. Table 1).

Collagen I immunoexpression in stage I granulomas was extracellular and fibrillar in the center of the lesion in lung (7/8; 87.5%) and mediastinal lymph nodes (3/3; 100%) (Fig. 1D). Collagen I immunoexpression was not observed in the periphery of the lesion in any stage I granuloma in lung (0/8; 0.0%) or mediastinal lymph nodes (0/3; 0.0%) (Table 2). Procollagen I immunoexpression was occasionally observed in the cytoplasm of epithelioid cells in some granulomas present in lung (6/8; 75%) and mediastinal lymph nodes (2/3; 66.7%) (Suppl. Table 1).

### Stage II granulomas

**Fig. 2:**
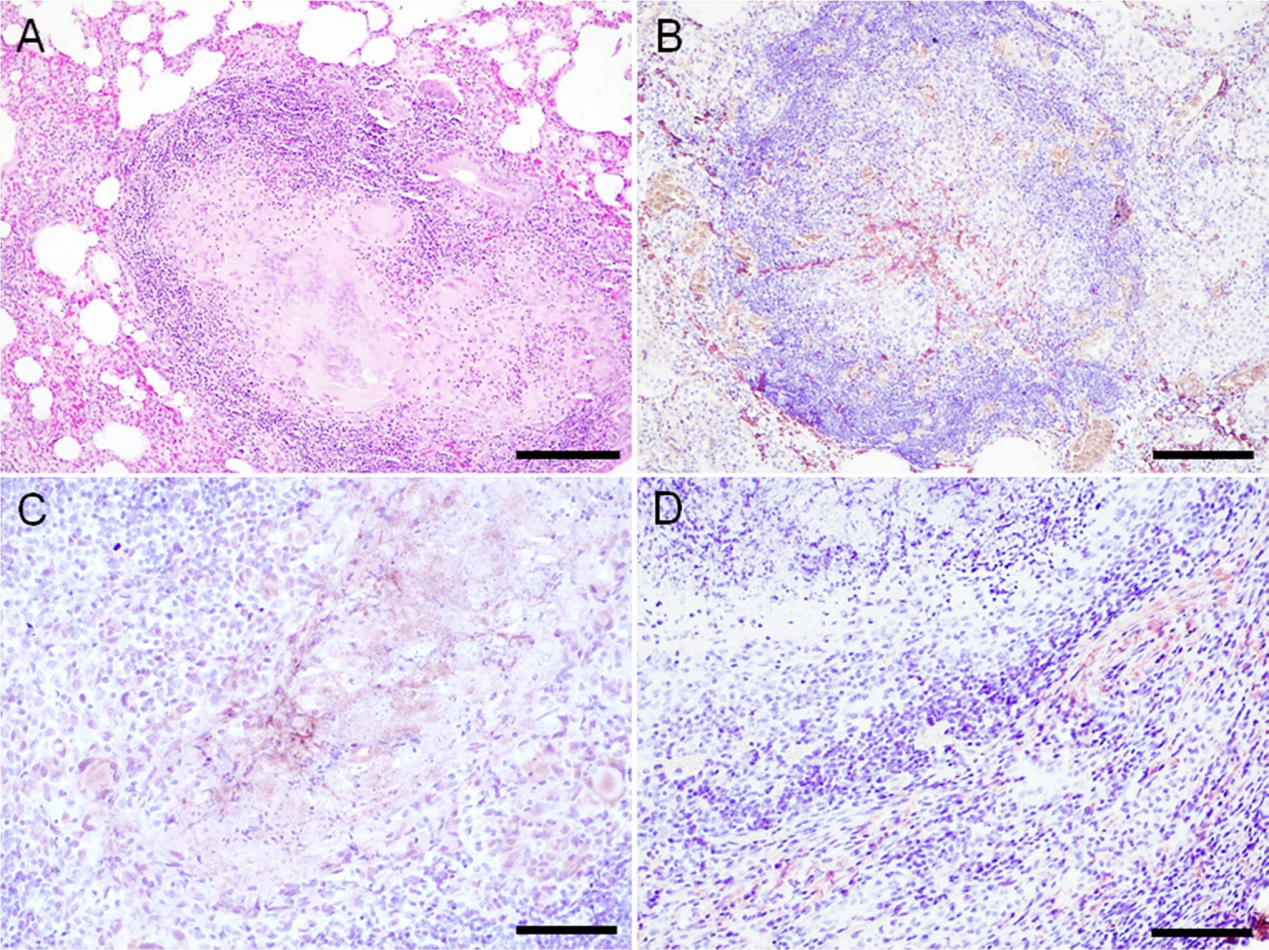
**A**-**D** Stage II granulomas (Wangoo et al. 2005) in goat (*Capra aegagrus hircus*) naturally infected with *Mycobacterium caprae*. **A**) Lung: chronic multifocal granulomatous pneumonia. HE, scale bar: 500 μm; **B**) Lung: extracellular, fibrillar-reticular, intense fibronectin immunoexpression in the centre and periphery of granulomas. Rabbit polyclonal anti-fibronectin antibody, scale bar: 500 μm; **C**) Lymph node: extracellular and cytoplasmic (epithelioid cells and Langhans-type multinucleated giant cells), moderate type III collagen immunoexpression in the centre and periphery of granulomas. Rabbit polyclonal anti-type III collagen antibody, scale bar: 200 μm; **D**) Lymph node: extracellular, fibrillar, mild type I collagen immunoexpression in the periphery of granulomas. Rabbit polyclonal anti- type I collagen antibody, scale bar: 200 μm

Histopathological study of the stage II granulomas (14/56; 25.0%), in both lung (8/14; 57.1%) and mediastinal lymph nodes (6/14; 42.9%) showed central necrosis surrounded by some epithelioid cells and Langhans-type multinucleated giant cells (Fig. 2A). Neutrophils and lymphocytes were also observed around the necrosis area. In addition, a thin connective tissue capsule was observed partially surrounding the lesions. Langhans-type multinucleated giant cells were large, round cells with ample pale eosinophilic cytoplasm and numerous nuclei located at the periphery of the cytoplasm in a “horseshoe” pattern.

Fibronectin immunoexpression was extracellular and fibrillar-reticular in the center of the lesions around the necrotic area in all stage II granulomas in lung (8/8; 100%) and in the majority of mediastinal lymph nodes (5/6; 83.3%) (Fig. 2B). Fibronectin immunoexpression was observed in the stage II granulomas in the periphery of the lesion in lung (3/8; 37.5%) and mediastinal lymph nodes (4/6; 66.7%) (Table 2). Cytoplasmic and diffuse, expression was observed in some epithelioid cells in some stage II granulomas in lung (3/8; 37.5%), but was not observed in mediastinal lymph node lesions (0/6; 0.0%). Intracytoplasmic and diffuse expression in fibroblasts was also observed in one of the lesions in lung (1/8; 12.5%) and mediastinal lymph nodes (1/6; 16.6%) (Suppl. Table 2).

Collagen III immunoexpression in stage II granulomas was extracellular and fibrillar in the center of the lesion, around the necrosis area, in lung (7/7; 100%) and mediastinal lymph nodes (5/5; 100%) (Fig. 2C). Collagen III immunoexpression in the periphery of stage II granulomas was extracellular and fibrillar in most lesions in lung (5/7; 71.4%) and mediastinal lymph nodes (5/5; 100%) (Table 2). Diffuse procollagen III expression in the cytoplasm of epithelioid cells and multinucleated Langhans-type giant cells was observed in lung (7/7; 87.5%) and mediastinal lymph node (5/5; 83.3%) granulomas (Suppl. Table 2).

Collagen I immunoexpression in stage II granulomas was extracellular and fibrillar in the center of the lesion around the necrosis area in lung (6/7; 85.7%) and mediastinal lymph nodes (5/6; 83.3%) (Fig. 2D). Collagen I immunoexpression in stage II granulomas was fibrillar in the periphery of the lesion in one case in lung (1/7; 14.3%) and more frequently in mediastinal lymph nodes (4/6; 66.7%) (Table 2). Diffuse procollagen I expression in the cytoplasm of some epithelioid cells was observed in some granulomas in lung (2/7; 28.6%) and mediastinal lymph nodes (1/6; 16.7%). The expression was not observed in the cytoplasm of fibroblasts in stage II lesions in lung (0/7, 0.0%), but in one case it was observed in mediastinal lymph node (1/6; 16.7%) (Suppl. Table 2).

### Stage III granulomas

**Fig. 3:**
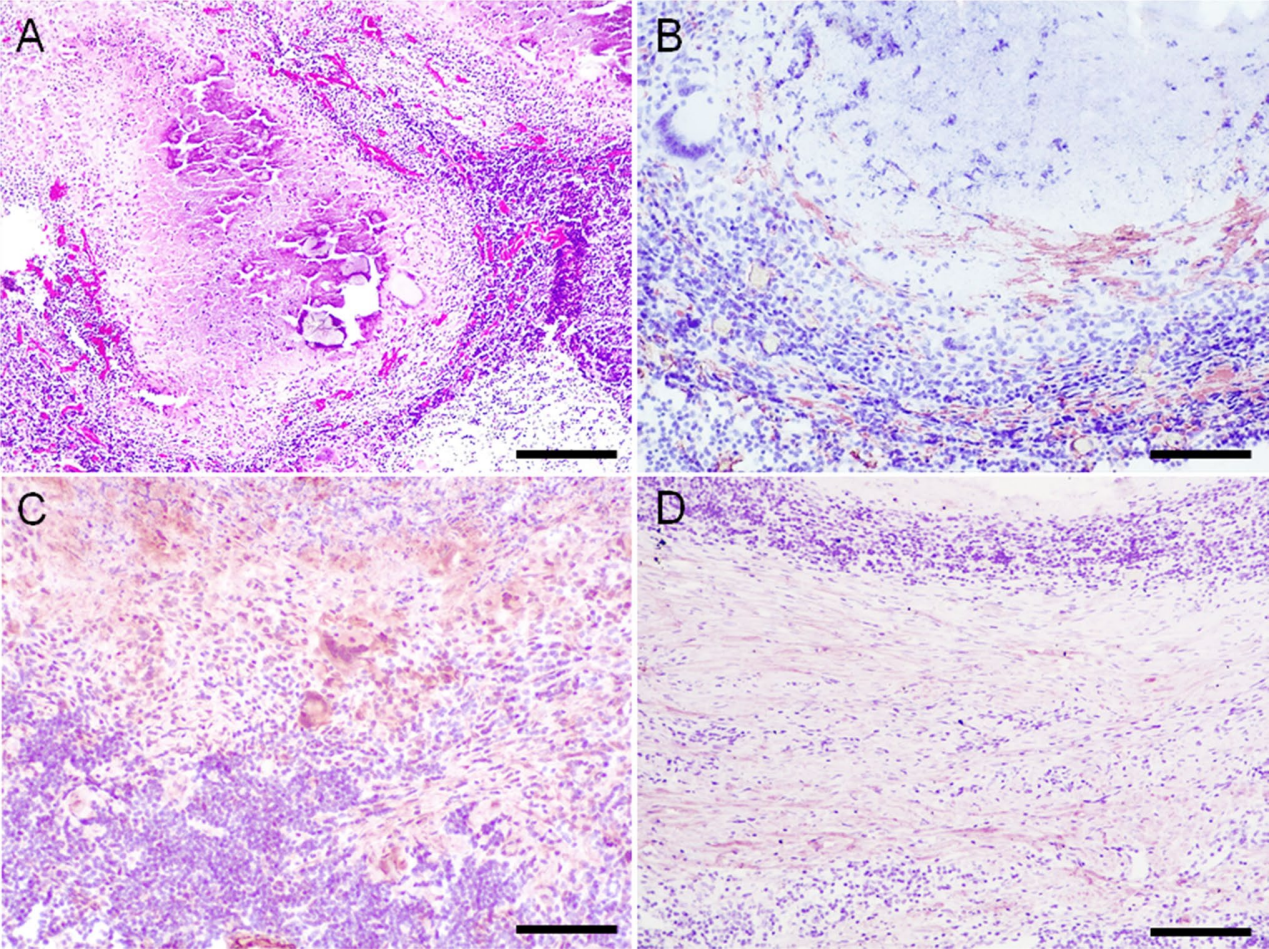
**A**-**D** Stage III granulomas (Wangoo et al. 2005) in goat (*Capra aegagrus hircus*) naturally infected with *Mycobacterium caprae*. **A**) Lymph node: chronic multifocal granulomatous lymphadenitis. HE, scale bar: 500 μm; **B**) Lymph node: extracellular, fibrillar, intense fibronectin immunoexpression in around the necrotic area and in the periphery of granulomas (double expression band). Rabbit polyclonal anti-fibronectin antibody, scale bar: 200 μm; **C**) Lymph node: extracellular and cytoplasmic (epithelioid cells, Langhans-type multinucleated giant cells and fibroblasts) moderate type III collagen immunoexpression. Rabbit polyclonal anti-type III collagen antibody, scale bar: 200 μm; **D**) Lymph node: extracellular, fibrillar, mild type I collagen immunoexpression. Rabbit polyclonal anti- type I collagen antibody, scale bar: 500 μm

Histopathological study of stage III granulomas (27/56; 48.2%) in lung (14/27; 51.9%) and mediastinal lymph nodes (13/27; 48.1%) showed extensive areas of central caseous necrosis with mineral deposits (dystrophic mineralization) surrounded by a dense layer of epithelioid cells and Langhans-type multinucleated giant cells, as well as occasional viable and degenerate neutrophils (Fig. 3A). Externally, a thin layer composed of lymphocytes and occasional plasma cells was observed. Numerous fibroblasts and parallel connective tissue fibers completely surrounded the lesion forming a capsule.

Fibronectin immunoexpression in stage III granulomas was extracellular and fibrillar-reticular in the center of the lesion surrounding the necrosis area in lung (13/14; 92.9%) and mediastinal lymph nodes (13/13; 100%) (Fig. 3B). At the periphery of the lesion, an extracellular and fibrillar fibronectin immunoexpression was observed in all stage III granulomas in both lung (14/14; 100%) and mediastinal lymph nodes (13/13; 100%) forming a double parallel band of fibronectin expression (Table 2). Intracytoplasmic and diffuse expression was observed in fibroblasts in stage III granulomas in lung (8/14; 57.1%) and mediastinal lymph nodes (6/13; 12.5%) Some epithelioid cells in one of the stage III granulomas in lymph node showed fibronectin immunoexpression (1/13; 7.7%) (Suppl. Table 3).

Collagen III immunoexpression in stage III granulomas was extracellular and fibrillar in the center of the lesion around the necrosis area in lung (6/9; 66.7%) and mediastinal lymph nodes (11/11; 100%) (Fig. 3C). In the periphery of the lesion, extracellular and fibrillar collagen III immunoexpression was observed in both lung (9/9; 100%) and mediastinal lymph nodes (11/11; 100%) forming a double parallel band of collagen III immunoexpression (Table 2). Diffuse procollagen III expression in the cytoplasm of multinucleated Langhans-type giant cells was observed in the granulomas present in lung (8/9; 88.9%) and mediastinal lymph nodes (9/11; 81.2%). Diffuse expression was also observed in the cytoplasm of fibroblasts in granulomas in lung (8/9; 88.9%) and mediastinal lymph nodes (11/11; 100%) (Suppl. Table 3).

Collagen I immunoexpression in stage III granulomas was extracellular and fibrillar in some granulomas in the center of the lesion around the necrosis area in lung (2/9; 22.2%) and mediastinal lymph nodes (2/12; 16.7%) (Fig. 3D). In the periphery of the granuloma, collagen I immunoexpression was extracellular and fibrillar in lung (9/9; 100%) and mediastinal lymph nodes (11/12; 91.7%) (Table 2). Procollagen I expression was observed in the cytoplasm of some fibroblasts in lesions in lung (4/9; 44.4%) and mediastinal lymph node (5/12; 41.7%) (Suppl. Table 3).

## Discussion

Fibronectin is an extracellular matrix protein with adhesion function that constitutes the scaffold for the deposition of collagens and proteoglycans in fibrosis (Jayasankar et al. 2002; Marshall et al. 1996). In this study, consistent expression of fibronectin was observed in the extracellular space, forming a network in the center of stages I, II and III tuberculous granulomas in lung and mediastinal lymph node. Fibronectin immunoexpression was exclusively central in stage I granulomas, suggesting that the cells that compose the granulomas in early stages participate in fibronectin synthesis. Different cells can play an important role in the fibrotic process in tuberculous granulomas, although their contribution is still unclear (DiFazio et al. 2016). In this study, cytoplasmic expression of fibronectin has been observed in stage II granulomas in the lung inside macrophages and was reduced in the cytoplasm of fibroblasts. Interestingly, in stage III pulmonary granulomas, fibronectin intracellular expression was observed mainly in fibroblasts, and also to a lesser extent in macrophages. These findings support the recent theory of macrophage-myofibroblast mesenchymal transition in tuberculous granuloma which suggests that some macrophages differentiate into myofibroblasts and synthesize extracellular matrix proteins for capsule formation (Evans et al. 2020). Some authors have also pointed out that the excessive presence of fibronectin in chronic lesions in human cutaneous TB is a consequence of its synthesis by macrophages and fibroblasts in previous stages (Marshall et al. 1996).

Central and peripheral immunoexpression of fibronectin was observed in stages II and III granulomas. Similarly, in human cutaneous TB, fibronectin immunoexpression has been observed in the periphery of granulomas (Marshall et al. 1996). The simultaneous central and peripheral expression that form a double parallel band is more evident and constant in stage III granulomas in both lung and lymph nodes. As fibronectin provides a favorable substrate for cell migration, the distribution observed here indicates a potential structural support for collagen deposition, suggesting active fibrogenesis (Marshall et al. 1996). Nevertheless, the wide distribution of fibronectin observed in tuberculous granulomas can be exploited by mycobacteria, which possess fibronectin-binding proteins (Bisht and Meena 2019). Therefore, fibronectin in tuberculous granulomas could facilitate tissue colonization, and therefore high fibronectin immunoexpression in tuberculous granulomas may be an unfavorable indicator of clearance capacity.

Fibroblasts and myofibroblasts are the main cells responsible for the fibrosis process in tuberculous granulomas by synthesizing extracellular matrix proteins, such as procollagen (Warsinske et al. 2017). In this study, collagen III immunoexpression was observed in the center and periphery of tuberculous granulomas. The expression of procollagen III around lung granulomas in humans has been considered weaker than collagen I immunoexpression (Kaarteenaho-Wiik et al. 2007). However, our findings showed that the expression of collagen III in granulomas was more intense than the expression of collagen I.

Here, higher collagen III expression has been observed in the center of stage I granulomas and residual expression in the periphery in some mediastinal lymph node granulomas as reported in minipigs (*S. scrofa domesticus*) used as a model of TB (Gil et al. 2010). Subsequently, in stage II granulomas, collagen III expression was maintained in the center and increased peripherally. Finally, the expression of collagen III in stage III granulomas slightly decreased in the center and increased in the periphery, and a double parallel band of immunoexpression was observed. The presence of central fibrosis has been related to sterility in tuberculous granulomas studied after treatment with antimicrobials (DiFazio et al. 2016; Warsinske et al. 2017). Here, the increased collagen III expression in goat granulomas suggests a possible indicator of infection clearance, but further studies analyzing mycobacteria viability are required to confirm this hypothesis.

The double band of collagen III expression suggests active fibrogenesis in lesions, as indicated elsewhere (Gil et al. 2010). The cytoplasmic expression of collagen III in macrophages (stage I and II granulomas) and Langhans-type multinucleated giant cells (stage I, II and III) is probably due to the phagocytosis of extracellular matrix proteins along with the mycobacteria. Still, the cytoplasmic immunoexpression of procollagen III in macrophages (stage I and II), Langhans-type multinucleated giant cells (stage I, II and III) and fibroblasts (stage III) may also suggest the participation of these cells in granuloma fibrogenesis, supporting the hypothesis of mesenchymal transition of macrophages in TB (Evans et al. 2020).

The most common extracellular matrix component is collagen I, which accounts for 84% of the collagen produced by fibroblasts (DiFazio et al. 2016). Collagen I immunoexpression was described as fibrillar in the center of stage I, II and III granulomas, and progressively decreasing in mature granulomas. The results described here contrast with studies indicating that procollagen I expression in stages I and II tuberculous granulomas is absent and minimal, respectively, in cattle lesions (Wangoo et al. 2005). Here, the collagen I immunoexpression was not observed in the periphery of stage I granulomas, which is explained by the absence of capsule in this early lesion (Wangoo et al. 2005). Some authors have described procollagen I expression in stage III granulomas around the necrotic areas forming the capsule in cattle (Wangoo et al. 2005). Likewise, we have observed a progressive loss of collagen immunoexpression in the center, and maintenance of the peripheral expression in stage III granulomas. Dual expression of procollagen I in the center and periphery of granulomas has previously been described in pulmonary and cutaneous TB in humans (Kaarteenaho-Wiik et al. 2007; Marshall et al. 1996). As the presence of collagen I indicates synthesis of new collagen (Kaarteenaho-Wiik et al. 2007; Wangoo et al. 2005), our results suggest fibrogenesis in stage III granulomas.

The synthesis of procollagen I in human pulmonary TB has been associated with the presence of an increased number of active myofibroblasts (Kaarteenaho-Wiik et al. 2007). Our results also suggest a predominantly peripheral activity of myofibroblasts that synthesize collagen I to form the capsule of stage III granulomas. This is supported by the collagen I expression in stage III granulomas, especially as procollagen in the cytoplasm of fibroblasts but also in macrophages and Langhans-type multinucleated giant cells. In macaques (*Macaca fascicularis*), collagen I expression in the outermost area of granulomas is mediated by interleukins 4, 13 and transforming growth factor β (TGF-β) (Evans et al. 2020), indicating that macrophages present in the lesion contribute to fibrogenesis by stimulating myofibroblast activity indirectly at the periphery of stage III granulomas. Procollagen I expression in tuberculous granulomas is associated with lesion development and could be used as a marker of progression and chronicity, as previously suggested (Wangoo et al. 2005). Finally, simultaneous collagen I and III expression has been observed in stage II and especially stage III granulomas in our study. The expression of both collagens I and III has been observed in human pulmonary TB indicating the complexity of the capsule in TB (Kaarteenaho-Wiik et al. 2007).

Our study has some limitations. First, the same granuloma was not evaluated for all antibodies iN some occasions as some lesions were absent because of microtome slicing during histological processing. Second, we were not able to determine the state of chronicity of the lesions as samples were from naturally-infected goats. Further studies comparing the pathology of *M. caprae*, *M. bovis*, *M. tuberculosis* in the goat are required to understand whether granuloma evolution is influenced by the MTC specie and to stablish a histopathological grading scheme for caprine tuberculous granulomas.

## Conclusion

Fibrogenesis in *M. caprae* tuberculous granulomas is a complex, dynamic and ongoing process resulting from the interaction between the pathogen and the host. The main extracellular matrix proteins fibronectin, collagen III, and collagen I are involved in the process from incipient lesions to the formation of a complete capsule around the lesion. Immunoexpression of these extracellular matrix proteins could contribute to understanding fibrogenesis and dating in tuberculous granuloma in both animal models and humans.

## Supplementary Information

ESM 1 (DOCX 23 kb)

ESM 2 (DOCX 21 kb)

ESM 3 (DOCX 23 kb)

Supplementary Figure 1 A-C: Negative controls. A) Rabbit polyclonal anti-fibronectin antibody, scale bar: 200 μm; B) Rabbit polyclonal anti-type III collagen antibody, scale bar: 200 μm; C) Rabbit polyclonal anti- type I collagen antibody, scale bar: 200 μm. (PNG 833 kb)

High Resolution Image (TIF 1162 kb)

## Data Availability

All data generated or analysed during this study are included in this published article.
