# Supplementary material for: Extracellular matrix proteins (fibronectin, collagen III, and collagen I) immunoexpression in goat tuberculous granulomas (Mycobacterium caprae)
Source: Vet Res Commun. 2022 Sep 22;46(4):1147–56. doi: 10.1007/s11259-022-09996-3 (PMC9684263; doi:10.1007/s11259-022-09996-3)
Supplement: Supplementary file 1 — (DOCX 23 kb) [file 11259_2022_9996_MOESM1_ESM.docx]

**Supp. Table 1**. Immunohistochemical results of fibronectin, type III collagen and type I collagen in stage I granulomas (Wangoo *et al*., 2005) in goat (*Capra aegagrus hircus*) naturally infected with *Mycobacterium caprae*.

| **Stage I** | **Fibronectin** | | | **Type III collagen** | | | **Type I collagen** | | |
| --- | --- | --- | --- | --- | --- | --- | --- | --- | --- |
| **Lung** | **Centre** | **Periphery** | **Cells** | **Centre** | **Periphery** | **Cells** | **Centre** | **Periphery** | **Cells** |
| No. 1 | + | - | - | + | - | - | + | - | EC |
| No. 2 | + | - | EC | + | - | EC, MGC | + | - | EC |
| No. 3 | + | - | - | + | - | EC, MGC | - | - | EC |
| No. 4 | + | - | EC | + | - | EC, MGC | + | - | - |
| No. 5 | + | - | EC | + | - | EC, MGC | + | - | - |
| No. 6 | + | + | - | + | - | EC, MGC | + | - | EC |
| No. 7 | + | - | - | + | - | EC, MGC | + | - | EC |
| No. 8 | + | - | - | + | - | EC, MGC | + | - | EC |
| **Lymph node** | **Centre** | **Periphery** | **Cells** | **Centre** | **Periphery** | **Cells** | **Centre** | **Periphery** | **Cells** |
| No. 1 | - | - | EC | * | * | * | * | * | * |
| No. 2 | + | - | - | + | - | EC, MGC | + | - | EC |
| No. 3 | + | - | - | * | * | * | * | * | * |
| No. 4 | + | - | EC | * | * | * | * | * | * |
| No. 5 | + | - | EC | + | + | EC, MGC | + | - | EC |
| No. 6 | + | - | - | + | + | EC, MGC | + | - | - |
| No. 7 | + | - | EC | * | * | * | * | * | * |
| EC: epithelioid cell; MGC: Langhans-type multinucleated giant cell; asterisk (*) represent absence of granuloma in tissue samples due to histological processing (microtome). | | | | | | | | | |
