# Supplementary material for: Extracellular matrix proteins (fibronectin, collagen III, and collagen I) immunoexpression in goat tuberculous granulomas (Mycobacterium caprae)
Source: Vet Res Commun. 2022 Sep 22;46(4):1147–56. doi: 10.1007/s11259-022-09996-3 (PMC9684263; doi:10.1007/s11259-022-09996-3)
Supplement: Supplementary file 3 — (DOCX 23 kb) [file 11259_2022_9996_MOESM3_ESM.docx]

**Supp. Table 3**. Immunohistochemical results of fibronectin, type III collagen and type I collagen in stage III granulomas (Wangoo *et al*., 2005) in goat (*Capra aegagrus hircus*) naturally infected with *Mycobacterium caprae*.

| **Stage III** | **Fibronectin** | | | **Type III collagen** | | | **Type I collagen** | | |
| --- | --- | --- | --- | --- | --- | --- | --- | --- | --- |
| **Lung** | **Centre** | **Periphery** | **Cells** | **Centre** | **Periphery** | **Cells** | **Centre** | **Periphery** | **Cells** |
| No. 1 | - | + | - | - | + | - | - | + | - |
| No. 2 | + | + | F | * | * | * | * | * | * |
| No. 3 | + | + | F | * | * | * | * | * | * |
| No. 4 | + | + | F | * | * | * | * | * | * |
| No. 5 | + | + | F | * | * | * | * | * | * |
| No. 6 | + | + | - | + | + | F, MGC | + | + | F |
| No. 7 | + | + | - | * | * | * | * | * | * |
| No. 8 | + | + | F | + | + | F, MGC | - | + | - |
| No. 9 | + | + | F | + | + | F, MGC | - | + | F |
| No. 10 | + | + | F | + | + | F, MGC | - | + | - |
| No. 11 | + | + | F | - | + | F, MGC | - | + | F |
| No. 12 | + | + | - | - | + | F, MGC | - | + | - |
| No. 13 | + | + | - | + | + | F, MGC | - | + | F |
| No. 14 | + | + | - | + | + | F, MGC | + | + | - |
| **Lymph node** | **Centre** | **Periphery** | **Cells** | **Centre** | **Periphery** | **Cells** | **Centre** | **Periphery** | **Cells** |
| No. 1 | + | + | EC, F | + | + | F | - | + | F |
| No. 2 | + | + | - | + | + | F | - | - | - |
| No. 3 | + | + | F | * | * | * | + | + | F |
| No. 4 | + | + | - | * | * | * | * | * | * |
| No. 5 | + | + | - | + | + | F, MGC | - | + | F |
| No. 6 | + | + | - | + | + | F, MGC | - | + | - |
| No. 7 | + | + | - | + | + | F, MGC | - | + | - |
| No. 8 | + | + | - | + | + | F, MGC | - | + | F |
| No. 9 | + | + | F | + | + | F, MGC | - | + | - |
| No. 10 | + | + | F | + | + | F, MGC | - | + | - |
| No. 11 | + | + | F | + | + | F, MGC | - | + | - |
| No. 12 | + | + | F | + | + | F, MGC | - | + | F |
| No. 13 | + | + | - | + | + | F, MGC | + | + | - |

| F: fibroblasts; MGC: Langhans-type multinucleated giant cell; asterisk (*) represent absence of granuloma in tissue samples due to histological processing (microtome). |
| --- |
