# Supplementary figures and images for: Extracellular matrix proteins (fibronectin, collagen III, and collagen I) immunoexpression in goat tuberculous granulomas (Mycobacterium caprae)
Source: Vet Res Commun. 2022 Sep 22;46(4):1147–56. doi: 10.1007/s11259-022-09996-3 (PMC9684263; doi:10.1007/s11259-022-09996-3)

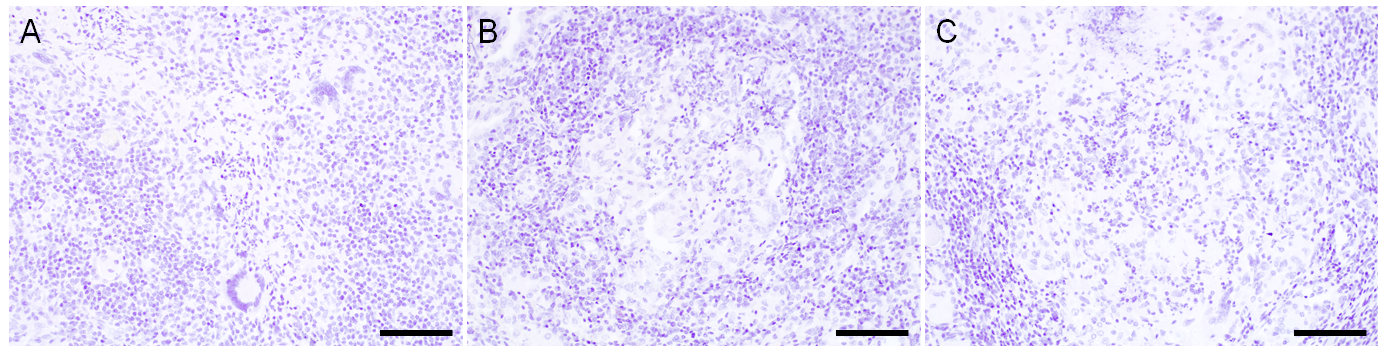

Supplement: Supplementary file 4 — A-C: Negative controls. A) Rabbit polyclonal anti-fibronectin antibody, scale bar: 200 μm; B) Rabbit polyclonal anti-type III collagen antibody, scale bar: 200 μm; C) Rabbit polyclonal anti- type I collagen antibody, scale bar: 200 μm. (PNG 833 kb) [file 11259_2022_9996_Fig4_ESM.png]

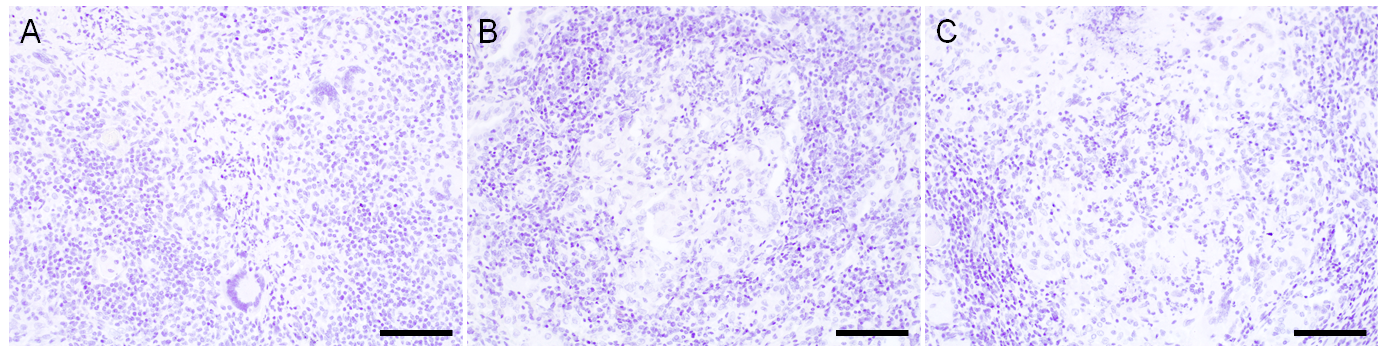

Supplement: Supplementary file 5 — High Resolution Image (TIF 1162 kb) [file 11259_2022_9996_MOESM4_ESM.tif]
